# Active withdrawal of corticosteroids using tocilizumab and its association with autoantibody profiles in relapsed Takayasu arteritis: a multicentre, single-arm, prospective study (the Ab-TAK study)

**DOI:** 10.3389/fimmu.2024.1473100

**Published:** 2025-01-07

**Authors:** Tsuyoshi Shirai, Tomonori Ishii, Soshi Okazaki, Yuko Shirota, Yusho Ishii, Hiroko Sato, Hiroshi Fujii

**Affiliations:** ^1^ Department of Clinical Rheumatology, Tohoku University Graduate School of Medicine, Sendai, Japan; ^2^ Department of Hematology and Rheumatology, Tohoku Medical and Pharmaceutical University, Sendai, Japan

**Keywords:** autoantibody, corticosteroid, Takayasu arteritis, tocilizumab, withdrawal

## Abstract

**Objectives:**

The feasibility of corticosteroid withdrawal (CW) for Takayasu arteritis (TAK) remains uncertain. Two autoantibodies (Abs) are identified against endothelial protein C receptor (EPCR) and scavenger receptor class B type 1 (SR-BI) in TAK, determining its three subgroups. This study aimed to evaluate CW using tocilizumab (TCZ) and its association with the Ab profile.

**Methods:**

This prospective study, lasted for 24 weeks, included patients with relapsed but stable TAK. Scheduled tapering of prednisolone (PSL) was performed with subcutaneous TCZ (CW at week 20). The primary endpoint was the difference in type A remission, defined by CW and the absence of inflammatory signs, according to the Ab profile at week 24.

**Results:**

Twenty patients were included and 18 patients with a mean PSL dose of 4.9 ± 2.8 mg/day were analysed. Anti-EPCR Ab-positive (E+), anti-SR-BI Ab-positive (S+), and double-negative (DN) groups included four (22.2%), eight (44.4%), and six (33.3%) patients, respectively. At week 24, the mean PSL dose was 2.0 ± 2.7 mg/day. Type A remission was observed in eight patients (44.4%), with significant differences based on the Ab profile: E+ (three patients, 75%), S+ (five patients, 62.5%), and DN (zero patients, 0%) (P=0.018). Besides, age, disease duration, PSL dose, type V arterial lesion, arterial dilation, and C-reactive protein >0.01 mg/dL were identified as risks for CW failure.

**Conclusion:**

CW using TCZ was achieved in 44.4% of patients with TAK relapse and was significantly higher in E+ and S+ patients. CW can be a feasible target, and the precise selection of patients is critical.

## Introduction

The application of molecular-targeted drugs in vasculitides has recently expanded. Large-vessel vasculitis, which comprises Takayasu arteritis (TAK) and giant cell arteritis (GCA), is also a disease for which the benefits of molecular-targeted drugs have been evaluated. These drugs include tocilizumab (TCZ), tumour necrosis factor (TNF) inhibitors, ustekinumab, abatacept, rituximab, and Janus kinase inhibitors (1–6). Their results showed a difference in response to treatment between TAK and GCA, indicating the importance of differentiating between these diseases. The effectiveness of TCZ in inhibiting interleukin (IL)-6 signalling in GCA was confirmed in a GiACTA study (2). Meanwhile, the primary endpoint was not met in TAK, although TCZ showed favorable time to relapse, which may have been affected by the small study sample size (7). Nonetheless, the effectiveness of TCZ in TAK has been recognised, and its use is recommended (8, 9). Additionally, significant effect of TCZ on reducing the dose of corticosteroid in TAK is reported in the real-world setting (10, 11). However, it remains unclear whether corticosteroid withdrawal (CW) using TCZ is a feasible target for patients with TAK.

The pathophysiology of TAK has not yet been fully elucidated. T cells are considered to be the key players, and myeloid cells including macrophages function as effector cells (12, 13). Additionally, recent evidence supports the contribution of B cells to the pathogenicity of TAK (14, 15), and two autoantibodies, anti-endothelial protein C receptor (EPCR) and anti-scavenger receptor class B type 1 (SR-BI) antibodies, were identified in TAK (16). These autoantibodies target protein receptors that play a role in resolving inflammation; therefore, they may be involved in the maintenance of vascular inflammation (16). A previous study suggested three TAK subgroups based on the presence of autoantibodies: anti-EPCR antibody-positive (E+), anti-SR-BI antibody-positive (S+), and double negative (DN). Analysis of the clinical features of these groups revealed relatively distinct phenotypes, suggesting the potential use of these autoantibodies for subgrouping TAK (16). In a previous study, the S+ group had wider vascular distribution and higher levels of inflammatory markers, while aortic regurgitation was less frequent. The E+ group had a higher incidence of cerebrovascular events and a limited number of vascular lesions. The surgical intervention and AR were higher in the DN group. Additionally, TAK is often accompanied by other autoimmune conditions (17–19), and a high frequency of ulcerative colitis (UC) has been observed in the E+ group (16). These results suggest different treatment responses among the subgroups.

Although corticosteroids are indispensable for the treatment of TAK, the lowest dose is preferable to avoid corticosteroid-induced toxicity in young patients, and CW is ideal. However, there is little evidence on whether CW is feasible for the treatment of TAK. Therefore, this pilot study prospectively evaluated the possibility of CW using TCZ in patients with TAK and its association with different subgroups defined by the autoantibody profile.

## Methods

### Patients

Participants who fulfilled the following five criteria were included: aged ≥20 years at the time of informed consent, diagnosis of TAK based on the Japanese Guidelines for Management of Vasculitis Syndrome (20) or the 1990 American College of Rheumatology classification criteria for TAK (21), absence of an inflammatory reaction, prednisolone (PSL) dose <20 mg/day, and previous relapse with >7.5 mg/day PSL. The required symptoms and findings for relapse were defined as the presence of at least one of the following: headache, dizziness, systemic symptoms (e.g., fever, fatigue, weight loss, joint symptoms, muscle symptoms), vascular lesions and associated symptoms, or persistent C-reactive protein (CRP) positivity. The study protocol complied with the principles of the Declaration of Helsinki and was approved by the Ethics Committee of the Tohoku University Graduate School of Medicine. All the patients provided written informed consent to participate in the study.

### Study design

This prospective, single-arm, multicentre trial was designed to evaluate the association between autoantibody profile and CW using TCZ in patients with TAK resistant to tapered corticosteroids. This study was conducted from July 2022 to December 2023. All included patients received weekly subcutaneous injections of 162 mg of TCZ. The total duration of this study was 24 weeks. The PSL dose was tapered according to the scheduled protocol, with CW implemented at week 20. The PSL dose tapered at each visit is detailed in **Supplementary Table 1**. If the maintenance of remission was difficult, corticosteroids were not withdrawn, and a sufficient dose was continued. This study aimed to compare three groups: E+, S+, and DN. Patients with double positivity for both autoantibodies were excluded, because double positivity is considered infrequent (16).

### Assessments

The primary endpoint was the difference in the rates of type A remission according to the autoantibody profile at week 24. Type A remission was defined by the CW and the absence of any signs suggestive of inflammation, systemic symptoms, laboratory data suggesting inflammation, new vascular lesions detected on physical examination, and new ischaemic events (**Supplementary Table 2**). Secondary endpoints included the following nine items (1): the difference in the rates of type C remission, defined by the absence of inflammatory signs and the dose of PSL ≤5 mg/day, according to the autoantibody profile at week 16; (2) difference in the rates of type B remission, defined by the absence of inflammatory signs and the dose of PSL ≤2.5 mg/day, according to the autoantibody profile at week 20; (3) difference in corticosteroid doses at relapse according to the autoantibody profile; (4) difference in the corticosteroid doses at week 24 according to the autoantibody profile; (5) disappearance of the symptoms at week 24 according to the autoantibody profile; (6) difference in the rates of relapse, initiation of immunosuppressive drugs, or change in the drugs for systemic symptoms at week 12, 16, 20, and 24 according to the autoantibody profile; (7) change in the laboratory data, including CRP, erythrocyte sedimentation rate (ESR), serum IL-6, and autoantibody titres; (8) change in computed tomography (CT) findings at week 24; and (9) safety profiles including adverse events, laboratory markers, and vital signs. Relapse was defined when two of the signs suggestive of inflammation are found.

### Measurement of autoantibodies

Serum samples were collected from all participants at the time of registration and week 24, and stored at -80°C until the measurement of autoantibodies. Autoantibody measurements were performed using a cell-based assay as described previously (16). Briefly, 1:10 diluted human serum was used as the primary antibody with 50 mg/mL goat gamma globulin fraction (Sigma-Aldrich), and a fluorescent-conjugated antibody was used as the secondary antibody. To quantify the activity, the relative mean fluorescence intensity (MFI) ratio was determined according to the following formula: (MFI of overexpressing cells - MFI of non-overexpressing cells)/MFI of non-overexpressing cells × 100. Cutoff values were predetermined as the mean + 3 standard deviations of the relative MFI ratio in healthy controls.

### Safety analysis

Adverse events were defined as those listed in the case report as Lowest Level Terms, Preferred Terms, or System Organ Class in the ICH International Glossary of Medical Terms Japanese Edition (MedDRA/J) version 26.1. In the summary report, the names of the adverse events were listed in the PT.

### Measurement of cytokine profile

Comprehensive analysis of cytokines at baseline and week 24 was performed using the Proteome ProfilerTM Array/Human XL Cytokine Array Kit (R&D Systems) according to the manufacturer’s instructions. Images were acquired using a FUSION Solo (Vilber).

### Patient and public involvement

Patients and the public were not involved in the design, conduct, reporting, or dissemination plans of this research.

### Statistical analysis

Primary efficacy analysis was performed using the full analysis set, and safety was assessed using the safety analysis set. An additional analysis was conducted using the per-protocol set to evaluate the sensitivity of the primary analysis. The primary and secondary endpoints were analysed using the Fisher’s exact, Kruskal–Wallis, or Mann–Whitney U test. SAS V. 9.4 (SAS Institute) was used as the statistical software.

## Results

### Patient characteristics

Twenty patients were enrolled in the study to analyse the safety of TCZ (**Supplementary Figure 1**). One patient dropped out because of aortic dissection, and 19 patients completed the study. One patient was excluded from the full analysis set because of their double positivity for anti-EPCR autoantibodies and anti-SR-BI autoantibodies. No patients were excluded from the per-protocol set, and 18 patients were analysed for full analysis set and per-protocol set, with clinical characteristics shown in **Table 1**. Of the 18 patients included in full analysis set and per-protocol set, 16 patients (89%) were female. The mean age and disease duration were 49.7 ± 15.3 and 18.7 ± 9.3 years, respectively. Co-existing UC was detected in one patient (5.6%). Polymyalgia rheumatica was absent. The mean dose of PSL was 4.9 ± 2.8 mg/day and all patients were on subcutaneous TCZ. Type V arterial lesions were observed in nine patients (50%). Stenotic and dilated arterial lesions were observed in 12 (66.7%) and nine (50.0%) patients, respectively (**Table 1**). The details of the vascular lesions detected using CT in this study are presented in **Supplementary Table 3**. Stenotic artery lesions were most frequent in the carotid artery (44.4%) and subclavian arteries (39%), followed by the renal artery and aortic arteries. In contrast, dilated arterial lesions were most common in the ascending aorta (27.8%), followed by the other parts of the aorta, and was less observed in the dilatation of the branches.

**Table 1:** Clinical characteristics of the patients.

Characteristic	Total	Anti-EPCR antibody positive	Anti-SR-BI antibody positive	Double negative
n(%)	18	4 (22.2)	8 (44.4)	6 (33.3)
Females (%)	16 (88.9)	4 (100.0)	7 (87.5)	5 (83.3)
Age, years	49.7 ± 15.3	47.5 ± 19.6	47.8 ± 17.9	53.7 ± 9.4
Disease duration, years	18.7 ± 9.3	19.2 ± 11.6	14.3 ± 7.0	24.2 ± 8.7
Weight, kg	56.5 ± 12.3	51.0 ± 4.5	56.6 ± 12.0	60.7 ± 16.1
Ulcerative colitis, n (%)	1 (5.6)	1 (25.0)	0 (0.0)	0 (0.0)
Polymyalgia rheumatica, n (%)	0 (0.0)	0 (0.0)	0 (0.0)	0 (0.0)
Prednisolone, mg/day	4.9 ± 2.8	5.0 ± 0.8	3.9 ± 3.9	6.2 ± 1.5
Tocilizumab	18 (100.0)	4 (100.0)	8 (100.0)	6 (100.0)
Vascular lesion n (%)				
I	2 (11.1)	0 (0.0)	2 (25.0)	0 (0.0)
IIa	2 (11.1)	2 (50.0)	0 (0.0)	0 (0.0)
IIb	3 (16.7)	0 (0.0)	1 (12.5)	2 (33.3)
III	2 (11.1)	0 (0.0)	1 (12.5)	1 (16.7)
IV	0 (0.0)	0 (0.0)	0 (0.0)	0 (0.0)
V	9 (50.0)	2 (50.0)	4 (50.0)	3 (50.0)
Stenotic arterial lesion	12 (66.7)	2 (50.0)	5 (62.5)	5 (83.3)
Dilated arterial lesion	9 (50.0)	2 (50.0)	2 (25.0)	5 (83.3)

Data are presented as number (percentage) or mean (standard deviation).

EPCR, endothelial protein C receptor; SR-B1, scavenger receptor class B type 1.

### Autoantibody positivity

The numbers of patients in the E+, S+, and DN groups were four (22.2%), eight (44.4%), and six (33.3%), respectively. One patient with UC belonged to the E+ group. Patients in the DN group tended to be older and had a higher body weight and longer disease duration. The E+ group had the most common type II arterial lesions, whereas type V arterial lesions were observed in half the patients in all groups. Stenotic and dilated arterial lesions were frequently observed in the DN group. Although the presence of stenotic lesions in the carotid and subclavian arteries was comparable between the groups, renal artery involvement was not observed in the E+ group. Dilatation of the ascending aorta was observed in 50% of patients in the DN group which was higher than that in the other groups.

### Primary endpoint

**Figure 1:**
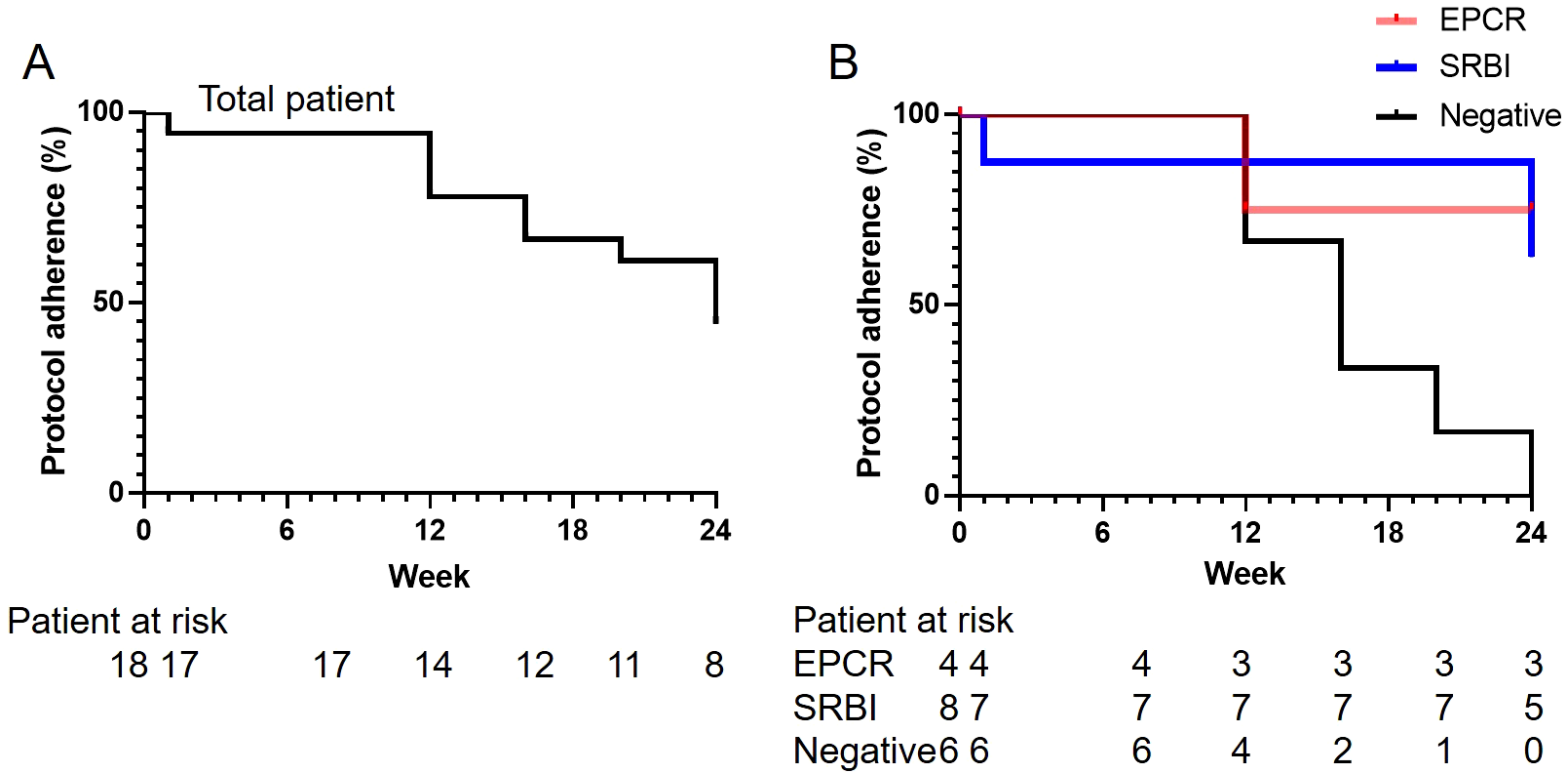
Rate of protocol adherence. The rates of protocol adherence **(A)** in the total population and **(B)** in patients with anti-EPCR antibody single positivity (EPCR), anti-SR-BI antibody single positivity (SR-BI), or autoantibody double-negativity (DN) are shown. EPCR, endothelial protein C receptor; SR-B1, scavenger receptor class B type 1.

The results of the primary endpoints are presented in **Table 2**. The type A remission (CW) rate (95% confidence interval [CI]), defined by CW and the absence of signs indicative of inflammation, at 24 weeks in the total, E+, S+, and DN groups were 44.4% (21.5%-69.2%), 75.0% (19.4%-99.4%), 62.5% (24.5%-91.5%), and 0.0% (0.0%-45.9%), respectively The achievement of type A remission (CW) was significantly higher in the E+ and S+ groups than in the DN group (P=0.018). Comparison of pairs between groups revealed statistically significant differences between the E+ and DN groups and the S+ and DN groups (P=0.033 and P=0.031, respectively). The rate of protocol adherence is shown in **Figures 1A, B**. None of the patients in the DN group were able to adhere to the protocol by the end of week 24 (**Figure 1B**). The reasons for the deviation from corticosteroid tapering are shown in **Supplementary Table 4**. Musculoskeletal symptoms were the most frequent (n=5) reason for the deviation from corticosteroid tapering, three cases of which were observed at week 24, four weeks after CW. Other reasons for the deviation from corticosteroid tapering included elevated IL-6 (n=3) and cutaneous manifestations (n=2). One patient complicated with UC in the E+ group presented with gastrointestinal symptoms.

**Table 2:** Primary endpoint: Achievement of type A remission.

	Total	Anti-EPCR antibody positive	Anti-SR-BI antibody positive	Double negative	P-value			
	n=18	G1, n=4	G2, n=8	G3, n=6	All	G1:G2	G1:G3	G2:G3
Number (%)	8 (44.4)	3 (75.0)	5 (62.5)	0 (0.0)	0.018	1.00	0.033	0.031
95% CI	21.5-69.2	19.4-99.4	24.5-91.5	0.0-45.9				

CI, confidence interval; G, group; EPCR, endothelial protein C receptor; SR-B1, scavenger receptor class B type 1.

### Secondary endpoints

None of the secondary endpoints showed significant differences among the three groups according to autoantibody profiles (**Supplementary Table 5**). The B remission (PSL ≤2.5 mg/day) rates and the C remission (PSL ≤5 mg/day) rates were comparable. Relapse was observed only in one patient in the S+ group at 2 mg/day of PSL. At 24 weeks, the mean dose of PSL was reduced to 2.0 ± 2.7 mg/day in the total population, and 1.3 ± 2.5, 1.8 ± 3.5, and 2.8 ± 1.9 mg/day in the E+, S+, and DN groups, respectively (P=0.14). At all assessment time points, from 12 to 24 weeks, none of the patients were administered additional immunosuppressive drugs. Changes in medications for systemic symptoms did not differ among the three groups. None of the patients showed a change in CT findings from baseline to week 24.

The changes in laboratory data are shown in **Table 3**. The median values of CRP and ESR values were limited to 0.010-0.015 mg/dL and 2.0-3.0 mm/hour, respectively. The median levels of serum IL-6 ranged 21.4-31.3 at baseline, while those at week 24 were 27.9, 27.4, and 61.6 pg/mL in the E+, S+, and DN groups, respectively. The median titre of anti-EPCR antibodies in the E+ group was 44.0 at baseline and 21.7 at week 24. The median titre of anti-SR-BI antibodies in the S+ group were 54.3 at baseline and 58.5 at week 24.

**Table 3:** Changes in inflammatory markers.

		Total	Anti-EPCR antibody positive	Anti-SR-BI antibody positive	Double negative
		n=18	n=4	n=8	n=6
CRP					
Baseline	Median (IQR)	0.010 (0.010, 0.020)	0.010 (0.010, 0.018)	0.010 (0.010, 0.010)	0.015 (0.010, 0.033)
	Range	0.01-0.06	0.01-0.02	0.01-0.06	0.01-0.04
Week 24	Median (IQR)	0.010 (0.010, 0.020)	0.010 (0.010, 0.018)	0.010 (0.010, 0.033)	0.010 (0.010, 0.025)
	Range	0.01-0.04	0.01-0.02	0.01-0.04	0.01-0.04
ESR					
Baseline	Median (IQR)	2.0 (2.0, 5.8)	2.0 (2.0, 11.8)	2.0 (2.0, 6.5)	2.5 (2.0, 5.8)
	Range	2-15	2-15	2-8	2-8
Week 24	Median (IQR)	2.0 (2.0, 3.3)	3.0 (2.3, 5.3)	2.0 (2.0, 5.0)	2.0 (2.0, 3.3)
	Range	2-12	2-6	2-12	2-4
Serum IL-6					
Baseline	Median (IQR)	27.9 (19.1, 62.0)	21.4 (10.1, 61.0)	31.3 (20.7, 54.3)	30.6 (22.1, 126.1)
	Range	8.8-206	8.8-71.7	17.0-78.9	15.6-206
Week 24	Median (IQR)	35.3 (23.0, 85.6)	27.9 (16.4, 37.0)	27.4 (16.8, 77.2)	61.6 (24.1, 230.8)
	Range	13.5-458	14.8-37.7	13.5-269	23.6-458
Autoantibody					
Baseline	Median	–	44.0	54.3	–
	IQR	–	27.6-63.1	50.3-60.0	–
Week 24	Median	–	21.7	58.5	–
	IQR	–	18.6-66.1	24.6-83.6	–

CRP, C-reactive protein; ESR, erythrocyte sedimentation rate; IQR, Interquartile range; EPCR, endothelial protein C receptor; SR-B1, scavenger receptor class B type 1.

### Safety

The analysis results for full analysis set (20 cases) are described below. The most common adverse events were arthralgia and dizziness in two patients each (10.0%). The most serious adverse events were bile duct stones and aortic dissection in one patient each (5.0%), both of which were grade 3 (severe), and the patient with aortic dissection was excluded from further analysis. In both cases, a causal relationship with the main study was ruled out and the outcome was mild. Nausea, gastroenteritis, leukopenia, and pruritus occurred in one patient each (5.0%), and the disease was non-severe. No defects were reported in the prefilled syringes or autoinjectors.

### Comparison of patients who achieved and did not achieve CW

Eight patients whose PSL dose was 0 mg at weeks 20 and 24 were grouped as those who achieved CW (CW+), and the other 10 patients were grouped as those with failed CW (CW-). The clinical characteristics of the CW+ and CW- groups are shown in **Table 4**. Because this investigation was exploratory, no statistical tests were performed. Both male patients belonged to the CW- group. CW- groups tended to have the following characteristics: older age, longer disease duration, higher body weight, and higher PSL dose. One patient with UC did not achieve CW because of intestinal symptoms. 60.0% of CW- possessed type V artery lesion, which was less frequent in CW+ (37.5%). Compared with stenotic arterial lesions, dilated arterial lesions tended to be more frequent in patients with CW-. The CRP levels were suppressed in all patients, with ranges of 0.01-0.01 and 0.01-0.06 mg/dL in CW+ and CW-, respectively; all patients with CRP >0.01 mg/dL did not achieve CW. Meanwhile, ESR and serum IL-6 levels were comparable. Three patients developed arthralgia after CW at week 20, and PSL was restarted at week 24.

**Table 4:** Baseline clinical characteristics of patients who achieved and did not achieve corticosteroid withdrawal.

	Corticosteroid withdrawal	
	Achieved n=8	Failed n=10
Females, n (%)	8 (100)	8 (80.0)
Age, years	44.4 ± 18.4	53.9 ± 11.6
Disease duration, years	16.6 ± 5.4	20.4 ± 11.5
Weight, kg	50.6 ± 5.0	61.3 ± 14.5
Ulcerative colitis, n (%)	0 (0.0)	1 (11.1)
Polymyalgia rheumatica, n (%)	0 (0.0)	0 (0.0)
Prednisolone, mg/day	3.1 ± 2.3	6.3 ± 2.5
Tocilizumab administration, n (%)	8 (100.0)	10 (100.0)
Vascular lesion, n (%)		
I	1 (12.5)	1 (10.0)
IIa	2 (25.0)	0 (0.0)
IIb	1 (12.5)	2 (20.0)
III	1 (12.5)	1 (10.0)
IV	0 (0.0)	0 (0.0)
V	3 (37.5)	6 (60.0)
Stenotic arterial lesion, n (%)	5 (62.5)	7 (70.0)
Dilated arterial lesion, n (%)	3 (37.5)	6 (60.0)
C-reactive protein (mg/dL)		
Median (IQR)	0.010 (0.010, 0.010)	0.015 (0.010, 0.033)
Range	0.01-0.01	0.01-0.06
ESR, mm/hour		
Median (IQR)	2.0 (2.0, 8.0)	2.0 (2.0, 3.5)
Range	2-15	2-8
Serum IL-6		
Median (IQR)	26.0 (15.4, 40.5)	30.6 (21.8, 84.0)
Range	8.8-58.8	15.6-206

ESR, erythrocyte sedimentation rate; IQR, Interquartile range; IL, interleukin.

### Serum cytokine profile

**Figure 2:**
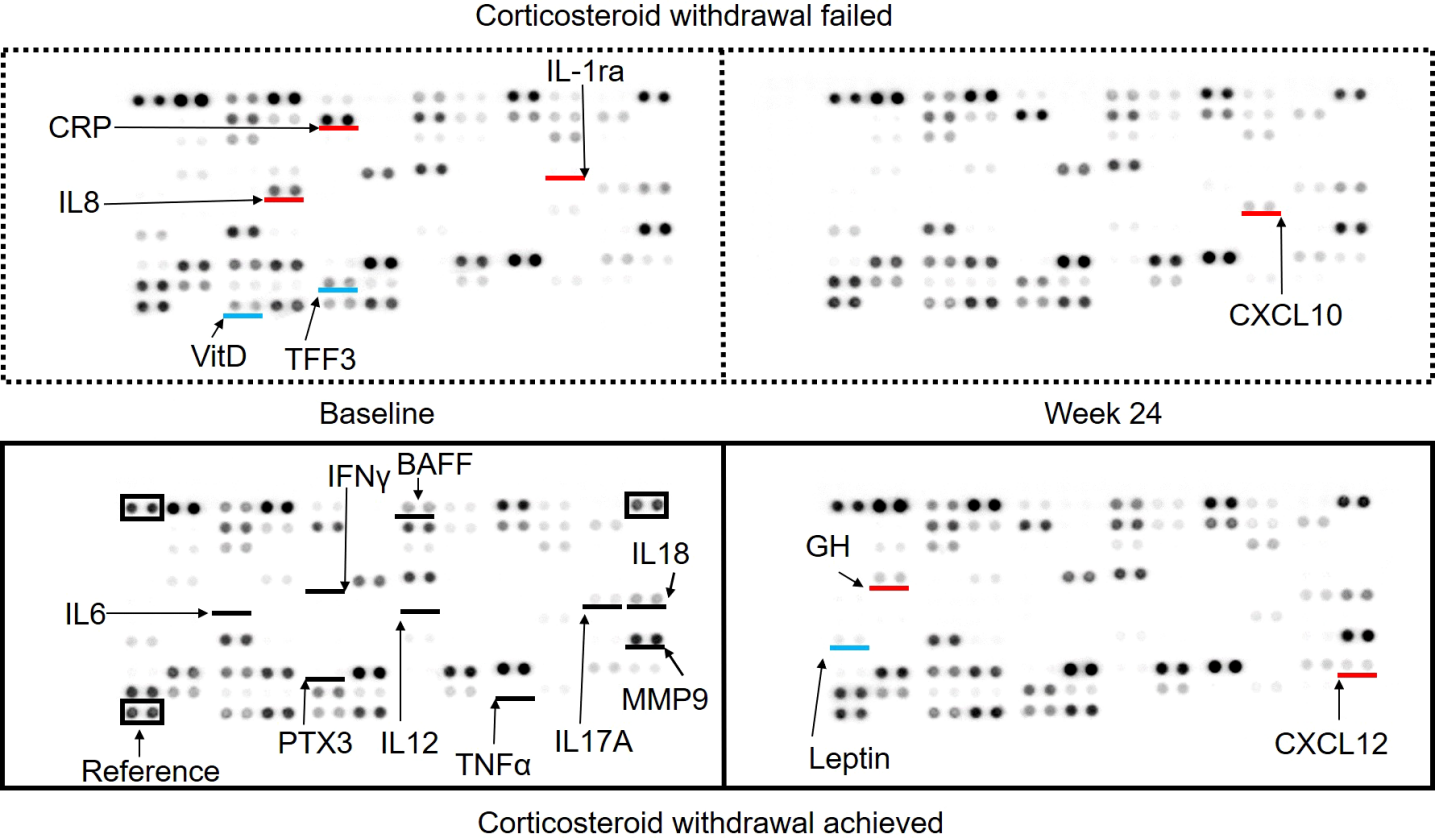
Serum cytokine array comparing patients who achieved and did not achieve corticosteroid withdrawal at baseline and week 24. The signals acquired using the cytokine profile kit are shown: upper left, baseline sera of patients with failed corticosteroid withdrawal (CW); upper right, week 24 sera of patients with failed CW; lower left, baseline sera of patients who achieved CW; and lower right, week 24 sera of patients who achieved CW. Black lines indicate the locations of specific cytokines reported to be involved in large-vessel vasculitis. Red and blue lines indicate cytokines with higher or lower expression compared to the baseline sera of patients who achieved corticosteroid withdrawal, respectively.

To elucidate whether specific cytokines were associated with CW, serum cytokines were compared among the CW+ and CW- groups using pooled sera at baseline and week 24, which were measured using a cytokine profile kit (**Figure 2**). In general, inflammatory cytokines, including IL-6, TNFα, and IL-12 were below the detection threshold in participants, while IL17A and IL18Bpa were detected, both of which were similar among groups. IL-8 was clearly detected in the baseline sera of the CW- group, and IL-1ra was weakly detected only in this group. The CRP signal was higher in the CW- group, whereas the PTX3 signal was comparable. In the baseline sera of the CW- group, TFF3 was less detected. A comparison of baseline and week 24 in the CW+ group showed a decrease in leptin and an increase in growth hormone and CXCL12 levels. Comparison of baseline and week 24 in the CW- group showed an increase in CXCL10 levels.

## Discussion

This prospective study provided several novel insights into TAK. First, an active corticosteroid tapering protocol using TCZ enabled CW in >40% of patients with TAK who relapsed. Second, the rate of CW in the absence of inflammatory signs was significantly different according to autoantibody profile, suggesting the importance of recognising subgroups in TAK. Third, the potent clinical features of patients achieving CW using TCZ were extracted. Furthermore, background information reminds us that vascular damage is still frequent (>50%) among patients with relapse, even in the modern era, and a long-term perspective balancing vascular damage and drug toxicity is required.

The difficulty in the management of TAK is its relapse rate (27–96%) (11, 22–27), Several factors are associated with relapse, such as initial monotherapy with a low dose of PSL (27) and a faster PSL dose reduction (24). In particular, the majority of relapses occurred during the tapering of PSL <20 mg/day (27). European League Against Rheumatism recommended tapering the doses of PSL to ≤10 mg/day for TAK and ≤5 mg/day for GCA after 1 year (8). The difference in the target dose was attributed to the high relapse rate of TAK when corticosteroids were substantially tapered. The American college of rheumatology (ACR) guidelines recommended CW in patients in remission; meanwhile, in patients with frequent relapse, continuation of corticosteroid for a longer duration was also noted (9). Members of the Japan Research Committee for Intractable Vasculitis also stated that more patients with TAK had difficulty tapering PSL than those with GCA (28). Based on the data of patients with TAK for >20 years, CW was possible in 30% of patients, and almost 20% achieved a drug-free status without the use of molecular-targeted drugs (11). However, the treatment of patients with relapse is challenging, and without the use of biologics, such patients require >10 mg/day of PSL, even when combined with other immunosuppressants (7, 11). Several studies have recently reported a tapered dose of PSL with biologics, which showed similar doses across studies: approximately 5 mg/day PSL (11, 29, 30). Harigai et al. conducted a phase 4, observational study to evaluate the effectiveness of TCZ in a real-world setting (29). In their study, CW was achieved in 42.5% and 13.7% of relapse-free patients in the initial and relapse treatment groups, respectively. The present study revealed that even higher rates (>40%) of CW could be achieved using an active tapering protocol in patients with relapse. It is still debatable whether TCZ should be used as a first-line therapy because some patients do not need biologics in the first place, and the assessment of disease activity becomes difficult with TCZ. Additionally, CW should be discussed together with long-term vascular damage, the evaluation of which requires several years to complete. The emergence of arthralgia following CW requires attention, and slowing down the tapering speed of PSL seems critical at low doses. Evaluation using positron emission tomography while attempting CW would be useful for detecting subclinical inflammation in patients receiving TCZ (10).

To elucidate the subpopulation in which TCZ can lead to CW, we focused on autoantibodies found in TAK. Although the existence and pathogenic functions of autoantibodies against endothelial cells have been recognised in TAK (15, 31), their target antigens are membrane components that are difficult to identify using conventional proteomic approaches (32). Using the serological identification system for autoantigens using a retroviral vector and flow cytometry method (32–34), EPCR and SR-BI membrane proteins expressed in the vasa vasorum of the aorta were identified as autoantigens (16). Autoantibodies against each autoantigen were found in about one-third of the patients, and three TAK subgroups based on the presence of autoantibodies were considered: E+, S+, or DN. Analysis of the clinical features of these groups revealed relatively distinct phenotypes as described in the Introduction. The frequent presence of anti-EPCR autoantibodies in TAK complicated with UC further led to the new knowledge that primary UC was another disease in which anti-EPCR antibodies were positive (16, 35, 36), which suggests that both TAK and UC share a similar underlying pathophysiology, for example, intestinal dysbiosis (37–39). Participants in this study presented similar clinical characteristics, while the frequency of type V arterial lesions in the DN group was higher than that in a previous report (16), suggesting that the presence of type V arterial lesions in the DN group was associated with a relapsed clinical course.

The primary endpoint, the difference in the rates of type A remission, defined by CW and the absence of inflammatory signs according to the profile of autoantibodies, was significantly different, and the absence of autoantibodies (i.e. the DN group) resulted in the failure of type A remission (CW). One patient in the E+ group with UC did not achieve CW because of intestinal symptoms. The efficacy of TCZ in UC has not been established (40); rather, TCZ might negatively affect intestinal inflammation (41, 42); therefore, other options, including TNF inhibitors, are preferred and probably better in the E+ group complicated with UC. The other patients in the E+ group were not complicated with UC, and all patients achieved type A remission (CW). The S+ group also showed a significantly higher rate of type A remission (CW) than the DN group, indicating that the presence of autoantibodies may be a predictive factor for CW in patients receiving TCZ.

The presence of autoantibodies indicated aberrant B cell activation in the E+ and S+ groups. IL-6 is a pleiotropic cytokine originally identified as a B-cell differentiation factor because it induces the maturation of B cells into antibody-producing cells (43). Blocking IL-6 signalling using TCZ leads to a significant shift in B cells from memory B cells to naïve B cells (44), which results in the inhibition of aberrant B cell activation. Additionally, anti-EPCR and anti-SR-BI antibodies are thought to play pathogenic roles in maintaining vascular inflammation (16). Therefore, TCZ diminishes the pathogenic function of autoantibodies. Nonetheless, these effects are in line with a successful report of rituximab in smouldering TAK, with multiple failures of molecular-targeted drugs (3). The levels of serum IL-6 tended to increase in the DN group during the tapering of corticosteroids, indicating the existence of subclinical inflammatory conditions (upstream of IL-6), which were suppressed by corticosteroids. Therefore, direct interventions are required for such upstream pathomechanisms in these patients. It should also be noted that patients in the DN group had a longer disease duration, which might have influenced their resistance to CW.

An exploratory investigation revealed the characteristics of patients who achieved CW using TCZ. In particular, male sex; older age; longer disease duration; higher body weight; higher dose of PSL; wider vascular lesions; presence of dilated arterial lesions; and CRP levels above the lowest value were indicative of patients who are resistant to treatment and overlapped with the characteristics observed in the DN group. One possible explanation is that the innate immune response is more prominent in these patients, whereas the adaptive immune response is more prominent in autoantibody-positive patients. This hypothesis is in line with the finding that IL-8 levels were high in the CW- group in cytokine measurement (**Figure 2**). Indeed, the use of TCZ made most of the inflammatory cytokines, including IL-6, IL-12, and TNFα, below the detection level of cytokine array. IL17A and IL18Bpa were detected and are potential targets for intervention. The CRP signal was higher in the CW- group, which was compatible with the data that higher levels of CRP were only detected in the CW- group (**Table 4**), whereas the signal of PTX3, which has been reported to correlate with disease activity in TAK (45), was not detected. Other slight changes in cytokines were also suggested among the samples at baseline and week 24, which reflected the influence of corticosteroids on leptin, growth hormone, and interferon. These observations require further validation by using a highly sensitive method for individual patients.

Regarding the safety of TCZ in TAK, infections and infestations are the most frequently reported (29, 30). Severe colitis and infective endocarditis are the severe complications of TCZ in patients with TAK (41, 42). However, no clinically problematic adverse events or illnesses related to TCZ were reported in this study.

This study had several limitations. First, this was a pilot study with a target of 20 patients, and a power analysis was not considered when setting the target number of cases. Since the patients were divided into three groups, a larger patient population is required for validation. Second, only a small number of cases were analysed in this study; however, the test results and P values are described as exploratory results. Third, information on previous treatments was not collected, which might have affected CW. Finally, the follow-up period was 24 weeks, and a longer duration is required to evaluate relapse and structural damage.

In conclusion, active CW using TCZ was achieved in 44.4% of the patients with TAK who experienced relapse. The autoantibody profile was associated with the achievement rate of CW with no inflammatory signs, which was significantly higher in the E+ and S+ groups than in the DN group. Other potent factors influencing CW were also recognised. A precise understanding of the subgroups of TAK would contribute to preventing toxicity caused by the long-term use of corticosteroids in a tailor-made manner.

## Data Availability

The original contributions presented in the study are included in the article/**Supplementary Material**. Further inquiries can be directed to the corresponding authors.
