# Supplementary material for: Active withdrawal of corticosteroids using tocilizumab and its association with autoantibody profiles in relapsed Takayasu arteritis: a multicentre, single-arm, prospective study (the Ab-TAK study)
Source: Front Immunol. 2025 Jan 7;15:1473100. doi: 10.3389/fimmu.2024.1473100 (PMC11747699; doi:10.3389/fimmu.2024.1473100)
Supplement: Supplementary file 1 [file DataSheet1.docx]

**SUPPLEMENTAL MATERIALS**

Active withdrawal of corticosteroids using tocilizumab and its association with autoantibody profiles in relapsed Takayasu arteritis: a multicentre, single-arm, prospective study (the Ab-TAK study)

Tsuyoshi Shirai*, Tomonori Ishii*, Soshi Okazaki, Yuko Shirota, Yusho Ishii, Hiroko Sato, Hiroshi Fujii

Corresponding author: Tsuyoshi Shirai MD, PhD and Tomonori Ishii MD, PhD

Tsuyoshi Shirai: tsuyoshi.shirai.d8@tohoku.ac.jp

Tomonori Ishii: tishii@med.tohoku.ac.jp


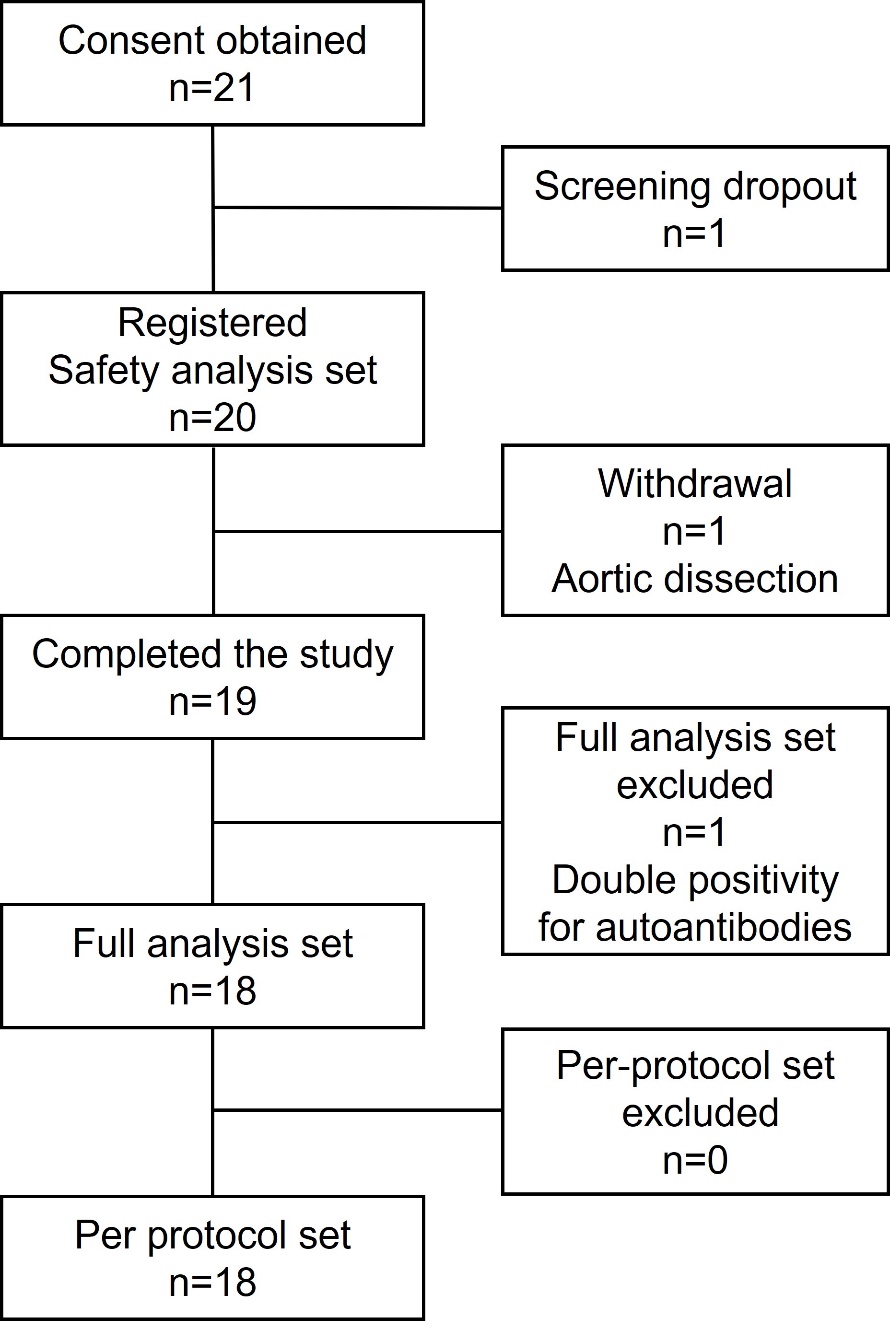


Supplemental Figure 1. Flow of patients in the study

Supplemental Table 1. Protocol of corticosteroid tapering

| VISIT | Baseline |  | 1 | 2 | 3 | 4 |  | 5 | 6 | 7 | 8 | 9 |
| --- | --- | --- | --- | --- | --- | --- | --- | --- | --- | --- | --- | --- |
| Week |  |  | 0 | 1 | 2 | 4 | 6 | 8 | 12 | 16 | 20 | 24 |
| Dose (PSL) | 20 |  | 17.5 | 15 | 12.5 | 10 | 8 | 6 | 4 | 2 | 0 | 0 |
|  | 17.5 - <20 |  | 17.5 | 15 | 12.5 | 10 | 8 | 6 | 4 | 2 | 0 | 0 |
|  | 15 - <17.5 |  | 15 | 15 | 12.5 | 10 | 8 | 6 | 4 | 2 | 0 | 0 |
|  | 12.5 - <15 |  | 12.5 | 12.5 | 12.5 | 10 | 8 | 6 | 4 | 2 | 0 | 0 |
|  | 10 - <12.5 | (n=1) | 10 | 10 | 10 | 10 | 8 | 6 | 4 | 2 | 0 | 0 |
|  | 9 - <10 |  | 9 | 9 | 9 | 9 | 8 | 6 | 4 | 2 | 0 | 0 |
|  | 8 - <9 | (n=2) | 8 | 8 | 8 | 8 | 8 | 6 | 4 | 2 | 0 | 0 |
|  | 7 - <8 |  | 7 | 7 | 7 | 7 | 7 | 6 | 4 | 2 | 0 | 0 |
|  | 6 - <7 | (n=3) | 6 | 6 | 6 | 6 | 6 | 6 | 4 | 2 | 0 | 0 |
|  | 5 - <6 | (n=5) | 5 | 5 | 5 | 5 | 5 | 5 | 4 | 2 | 0 | 0 |
|  | 4 -<5 | (n=3) | 4 | 4 | 4 | 4 | 4 | 4 | 4 | 2 | 0 | 0 |
|  | 3 -<4 | (n=1) | 3 | 3 | 3 | 3 | 3 | 3 | 3 | 2 | 0 | 0 |
|  | 2 -<3 | (n=1) | 2 | 2 | 2 | 2 | 2 | 2 | 2 | 2 | 0 | 0 |
|  | 1 - <2 |  | 1 | 1 | 1 | 1 | 1 | 1 | 1 | 1 | 0 | 0 |
|  | 0 | (n=2) | 0 | 0 | 0 | 0 | 0 | 0 | 0 | 0 | 0 | 0 |

PSL, prednisolone

Supplemental Table 2. The definition of remission in this study

|  | Dose of prednisolone | Week | Signs suggestive of inflammation |
| --- | --- | --- | --- |
| Type A remission | 0 | 24 | Absent |
| Type B remission | ≤2.5 mg/day | 20 | Absent |
| Type C remission | ≤5 mg/day | 16 | Absent |

Signs suggestive of inflammation: systemic symptoms, laboratory data suggesting inflammation, new vascular lesions detected on physical examination, and new ischaemic events

Supplemental Table 3. Vascular lesions detected in computed tomography

| Lesion | Total | Anti-EPCR antibody positive | Ant-SR-BI antibody positive | Double negative |
| --- | --- | --- | --- | --- |
|  | n=18 | n=4 | n=8 | n=6 |
| Stenosis |  |  |  |  |
| Carotid | 8 (44.4) | 2 (50.0) | 3 (37.5) | 3 (50.0) |
| Subclavian | 7 (39.0) | 1 (25.0) | 3 (37.5) | 3 (50.0) |
| Vertebral | 0 (0.0) | 0 (0.0) | 0 (0.0) | 0 (0.0) |
| Axillary | 0 (0.0) | 0 (0.0) | 0 (0.0) | 0 (0.0) |
| Pulmonary | 0 (0.0) | 0 (0.0) | 0 (0.0) | 0 (0.0) |
| Renal | 3 (16.7) | 0 (0.0) | 2 (25.0) | 1 (16.7) |
| Brachiocephalic | 1 (5.6) | 1 (25.0) | 0 (0.0) | 0 (0.0) |
| Aortic arch | 0 (0.0) | 0 (0.0) | 0 (0.0) | 0 (0.0) |
| Ascending | 1 (5.6) | 0 (0.0) | 0 (0.0) | 1 (16.7) |
| Descending | 2 (11.1) | 0 (0.0) | 1 (12.5) | 1 (16.7) |
| Abdominal | 2 (11.1) | 0 (0.0) | 1 (12.5) | 1 (16.7) |
|  |  |  |  |  |
| Dilatation |  |  |  |  |
| Carotid | 0 (0.0) | 0 (0.0) | 0 (0.0) | 0 (0.0) |
| Subclavian | 1 (5.6) | 0 (0.0) | 0 (0.0) | 1 (16.7) |
| Vertebral | 1 (5.6) | 1 (25.0) | 0 (0.0) | 0 (0.0) |
| Axillary | 0 (0.0) | 0 (0.0) | 0 (0.0) | 0 (0.0) |
| Pulmonary | 0 (0.0) | 0 (0.0) | 0 (0.0) | 0 (0.0) |
| Renal | 0 (0.0) | 0 (0.0) | 0 (0.0) | 0 (0.0) |
| Brachiocephalic | 1 (5.6) | 0 (0.0) | 1 (12.5) | 0 (0.0) |
| Aortic arch | 3 (16.7) | 1 (25.0) | 1 (12.5) | 1 (16.7) |
| Ascending | 5 (27.8) | 1 (25.0) | 1 (12.5) | 3 (50.0) |
| Descending | 2 (11.1) | 1 (25.0) | 0 (0.0) | 1 (16.7) |
| Abdominal | 2 (11.1) | 1 (25.0) | 1 (12.5) | 0 (0.0) |

Data are presented as number (percentage).

EPCR, endothelial protein C receptor; SR-B1, scavenger receptor class B type 1

Supplemental Table 4. Reason for deviating from corticosteroid tapering

| Profile | Week | Reason |
| --- | --- | --- |
| Anti-EPCR antibody positive | 12 | Gastrointestinal manifestation, diarrhea |
| Anti-SR-BI antibody positive | 1 | Damaged vascular lesion, elevated IL-6 |
| Anti-SR-BI antibody positive | 24 | Musculoskeletal symptom, arthritis |
| Anti-SR-BI antibody positive | 24 | Musculoskeletal symptom, arthralgia |
| Double negative | 12 | Elevated IL-6; cutaneous symptom, eruption |
| Double negative | 12 | Elevated IL-6 |
| Double negative | 16 | Cutaneous symptom, pruritis |
| Double negative | 16 | Musculoskeletal symptom, joint swelling |
| Double negative | 20 | Musculoskeletal symptom, back pain |
| Double negative | 24 | Musculoskeletal symptom, arthralgia |

EPCR, endothelial protein C receptor; SR-B1, scavenger receptor class B type 1; IL, interleukin

Supplemental Table 5. Secondary endpoints

| Secondary endpoint |  | Total | Anti-EPCR antibody positive | Anti-SR-BI antibody positive | Double negative |  |
| --- | --- | --- | --- | --- | --- | --- |
|  |  | n=18 | n=4 | n=8 | n=6 | P-value |
| B remission | Number (%) | 10 (55.6) | 3 (75.0) | 5 (62.5) | 2 (33.3) | 0.57 |
|  | 95% CI | 30.8-78.5 | 19.4-99.4 | 24.5-91.5 | 4.3-77.7 |  |
| C remission | Number (%) | 15 (83.3) | 3 (75.0) | 7 (87.5) | 5 (83.3) | 1.00 |
|  | 95% CI | 58.6-96.4 | 19.4-99.4 | 47.3-99.7 | 35.9-99.6 |  |
| PSL dose | at relapse | 2 | N/A | 2 | N/A | N/A |
|  | at 24 weeks | 2.0±2.7 | 1.3±2.5 | 1.8±3.5 | 2.8±1.9 | 0.14 |
| Rate of relapse | Number (%) | 1 (5.6) | 0 (0) | 1 (12.5) | 0 (0) | 1.00 |
|  | 95% CI | 0.1-27.3 | 0-60.2 | 0.3-52.7 | 0-45.9 |  |
| Disappearance of symptoms | Number (%) | 17 (94.4) | 4 (100) | 7 (87.5) | 6 (100) | 1.00 |
|  | 95% CI | 72.7-99.9 | 39.8-100 | 47.3-99.7 | 54.1-100 |  |
| Change in medication for systemic symptoms |  |  |  |  |  |  |
| 12 weeks | Number (%) | 1 (5.6) | 0 (0.0) | 1 (12.5) | 0 (0.0) | 1.00 |
|  | 95% CI | 0.1-27.3 | 0.0-60.2 | 0.3-52.7 | 0.0-45.9 |  |
| 16 weeks | Number (%) | 2 (11.1) | 1 (25.0) | 0 (0.0) | 1 (16.7) | 0.29 |
|  | 95% CI | 1.4-34.7 | 0.6-80.6 | 0.0-36.9 | 0.4-64.1 |  |
| 20 weeks | Number (%) | 2 (11.1) | 1 (25.0) | 0 (0.0) | 1 (16.7) | 0.29 |
|  | 95% CI | 1.4-34.7 | 0.6-80.6 | 0.0-36.9 | 0.4-64.1 |  |
| 24 weeks | Number (%) | 0 (0.0) | 0 (0.0) | 0 (0.0) | 0 (0.0) | N/A |
|  | 95% CI | 0.0-18.5 | 0.0-60.2 | 0.0-36.9 | 0.0-45.9 |  |
| Change in imaging | Number (%) | 0 (0) | 0 (0) | 0 (0) | 0 (0) | N/A |
|  | 95% CI | 0-18.5 | 0-60.2 | 0-36.9 | 0-45.9 |  |

EPCR, endothelial protein C receptor; SR-B1, scavenger receptor class B type 1; CI, confidence interval
